# Supplementary material for: A randomised experiment of health, cost and social norm message frames to encourage acceptance of swaps in a simulation online supermarket
Source: PLoS One. 2021 Feb 17;16(2):e0246455. doi: 10.1371/journal.pone.0246455 (PMC7888673; doi:10.1371/journal.pone.0246455)
Supplement: S1 Table — (DOCX) [file pone.0246455.s001.docx]

S1 Table. Results tables for linear regression models on energy change scores for total shopping baskets.

|  | *b (SE)* | *t* | *p* |
| --- | --- | --- | --- |
| **Unadjusted model** |  |  |  |
| Frame (Health frame as baseline) |  |  |  |
| Cost frame | 10.00 (7.46) | 1.34 | .181 |
| Social norm frame | -1.44 (7.44) | -0.19 | .847 |
| **Adjusted model** |  |  |  |
| Frame (health frame as baseline) |  |  |  |
| Cost frame | 9.80 (7.48) | 1.31 | .190 |
| Social norm frame | -1.49 (7.45) | -0.20 | .842 |
| Age | 0.44 (0.21) | 2.12 | .034 |
| Gender (female as baseline) | -16.57 (6.41) | -2.59 | .010 |
| BMI | -0.10 (0.49) | -0.20 | .845 |
| Education (Bachelor’s degree or equivalent as baseline) |  |  |  |
| None | -17.60 (20.45) | -0.86 | .390 |
| 4 GCSEs or fewer | -12.10 (10.21) | -1.19 | .236 |
| 5 GCSEs or more | -5.32 (9.37) | -0.57 | .571 |
| 2 A Levels or equivalent | 2.99 (8.72) | 0.34 | .732 |
| Post-graduate degree | 6.05 (9.68) | 0.63 | .532 |
| Income (Less than £15,5000 as baseline) |  |  |  |
| £15,500 - £24,999 | 8.55 (10.06) | 0.85 | .396 |
| £25,000-£39,999 | 10.67 (9.47) | 1.13 | .260 |
| More than £40,000 | 10.79 (9.47) | 1.14 | .255 |
