# Supplementary material for: A randomised experiment of health, cost and social norm message frames to encourage acceptance of swaps in a simulation online supermarket
Source: PLoS One. 2021 Feb 17;16(2):e0246455. doi: 10.1371/journal.pone.0246455 (PMC7888673; doi:10.1371/journal.pone.0246455)
Supplement: S1 File — (PDF) [file pone.0246455.s002.pdf]

Consent

Make sure you have 40 minutes available before starting the task. This is how long the task will take, and it should be completed in one go.

This survey is a research project being run by scientists within the University of Cambridge and from Public Health England (Study Information Sheet). If you have any questions please contact the researcher.

In summary you will be asked to:
- answer a few simple questions about you. No questions ask your name or other personal information.
- have a paper and pen handy to make a note of a shopping list.
- complete a food shopping exercise using our supermarket website.
- answer a few more questions about the shopping exercise

Give your consent to take part by confirming you understand the following:

- [ ] 1. I have read the Information for Participants and have had the opportunity to ask questions.
2. My participation is totally voluntary. I am free to withdraw at any time without having to give a reason.
3. All personal information will remain confidential to ensure I cannot be identified.
4. My data will be stored anonymously and securely, and may be used for future research.
5. I agree to take part in the Food Choices Study.
- [ ] OR **I do NOT agree to take part in this research study. Selecting this option will return me to the panel website**

Quality warning

Important – data quality!

Data quality is of the utmost importance for this survey. Please be aware that there are simple test questions in the survey that check whether you are reading the questions carefully. If you answer one of these test questions incorrectly, you will fail the quality control and will not proceed with the study.

- OK. I will read the questions carefully and am happy to proceed with the study.

Personal Info 1

Browser Meta Info

This question will not be displayed to the recipient.
Browser: **Firefox**
Version: **40.0**
Operating System: **Windows NT 6.1**
Screen Resolution: **1920x1080**
Flash Version: **18.0.0**
Java Support: **1**
User Agent: **Mozilla/5.0 (Windows NT 6.1; WOW64; rv:40.0) Gecko/20100101 Firefox/40.0**

Thinking about food shopping, which of these best describes the level of responsibility you have for the shopping in your household?

- Responsible for all or most of the food/grocery shopping
- Responsible for about half of the food/grocery shopping
- Responsible for less than half of the food/grocery shopping
- Not responsible for any of the food/grocery shopping

Do you sleep more than an hour (60 minutes) in total per night?

1 (Never)	2	3	4	5	6	7 (Always)
○	○	○	○	○	○	○

How often on average over the past year have you bought each of the following shopping list items for you or your household?

	Never or not in the last year	1-3 times in the last year	4-11 times in the last year	1-3 times a month	Once a week	2-4 times a week	5 or more times a week
- A loaf of bread	○	○	○	○	○	○	○
- Soup for a light meal	○	○	○	○	○	○	○
- Ready-to-eat meat or fish, or a packet of cheese	○	○	○	○	○	○	○
- Sweet biscuits	○	○	○	○	○	○	○
- Crisps or savoury snacks	○	○	○	○	○	○	○
- Chilled dairy desserts	○	○	○	○	○	○	○
- Meat or fish for a roast lunch or a vegetarian alternative	○	○	○	○	○	○	○
- A pack of pasta/rice/couscous /polenta or other starchy food	○	○	○	○	○	○	○
- A side dish to have with a main meal	○	○	○	○	○	○	○
- A snack to have between meals	○	○	○	○	○	○	○
- A sandwich filling	○	○	○	○	○	○	○
- A treat for you to eat straight away	○	○	○	○	○	○	○

How often have you been to every country in the world (all 196 of them).

Never	Not in the past year	1-3 times a year	4-11 times a year	1-3 times a month	Once a week	More than once a week.
○	○	○	○	○	○	○

Your gender

○ Male

○ Female

Your age (years)

[ ]

What is the highest education qualification you have achieved?

- None
- Up to 4 GCSE's (Including 1-4 O Levels/CSE/GCSEs (any grades), Foundation Diploma, NVQ level 1, Foundation GNVQ or equivalents)
- 5 or more GCSE's or 1 A-level (Including 5+ GCSEs (Grades A*-C),1 A Level/ 2-3 AS Levels, NVQ level 2, Intermediate GNVQ, City and Guilds Craft, BTEC First/General Diploma, RSA Diploma, Apprenticeship or equivalents)
- 2 or more A-levels (Including 2+ A Levels, 4+ AS Levels, NVQ Level 3, Advanced GNVQ, City and Guilds Advanced Craft, ONC, OND, BTEC National, RSA Advanced Diploma or equivalents)
- Bachelor's degree (Including BA, BSc, NVQ Level 4-5, HNC, HND, RSA Higher Diploma, BTEC Higher level or equivalents)
- Post-Graduate degree or qualification (Including Higher Degrees e.g. MA, PhD, PGCE, Professional qualifications e.g. teaching, nursing, accountancy or equivalents)

Shopping task

You are now going to complete a food shopping exercise using an online supermarket website. This is not a real commercial site, and you will not be asked to spend you own money or enter any personal details.

Have a pen and paper ready. You will need to make a note of your shopping list.

Click Next when you are ready.

Thank you for completing the shopping task.

How did you get on?

If you managed to buy everything on the list, please click '>>' below to finish the survey.

If you didn't quite follow the list, please have another go at the shopping task. Make a note of the list again and click on the link below. You will return to the supermarket to start again (your basket will be empty). We will use the data from your shopping task that follows the list the closest.

Imagine you are about do a real shopping task using your own money. Please can you 'buy' all the foods listed below using our online supermarket website. When you have chosen all the items on the list, check-out from the supermarket to bring you back to this survey and finish the study.

Please choose foods you would actually buy for you or your household. The budget is a guideline amount – you do not need to spend it all, but please try to stay within it.

Please now write down the list to make sure you get everything. Then click the link to the supermarket.

Your budget: £25

Your shopping list:

- A loaf of bread (approx 800g)
- Soup for a light meal for one
- A packet of ready-to-eat meat or fish, or a packet of cheese.
- A pack of sweet biscuits
- Crisps or savoury snacks for 6 people
- A chilled dairy-based dessert for 6 people
- Meat/ fish/ vegetarian alternative for a roast lunch (approx. 500g)
- 500g pack of pasta or rice or couscous or polenta or other starchy food
- A side dish to have with a main meal
- A snack to have between meals
- A sandwich filling
- A treat for you to eat straight away.

Click below to access the supermarket and select all the items on the list. Remember to checkout when you have finished to return to this page.

Visit the Supermarket website

Post study questions

Overall, how would you rate your shopping experience using Woods, our online supermarket website?

- Excellent
- Very Good
- Good
- Fair
- Poor

The online supermarket you have just used offered you alternative foods to some of the foods you originally chose. Is this something that you would like to have when you do your usual shopping?

- Very strongly like
- Strongly like
- Somewhat like
- Indifferent
- Somewhat dislike
- Strongly dislike
- Very strongly dislike
- I didn't notice any alternatives being offered to me.

How often, on average over the past year, have you..

	Never or not in the last year	1-3 times in the last year	4-11 times in the last year	1-3 times a month	At least once a week
... shopped online for food or groceries to be delivered to you (e.g. Tesco.com, Ocado.com, mysupermarket.co.uk)?					
... shopped online for non-food items to be delivered to you (e.g. books, clothes, electronics)?					

What units do you prefer to use to tell us your weight and height?

	Metric (kg, m, cm)	Imperial (stone, lb, feet, inches)
Weight		
Height		

Your height (metric):

Centimeters

Your height (imperial):

Feet	Inches

Your weight (metric):

kg

Your weight (imperial):

stone	lb

What is your total household income per year before tax (also known as gross per annum salary)

- Less than £15,500
- Between £15,500 and £24,999
- Between £25,000 and £39,999
- More than £40,000

You have now completed the study.

Many thanks for taking part.

Clicking 'next' will take you back to the recruitment agency website.

Please tell us if you have any comments or anything you think we should know about your experience of taking part in this study. (optional)

Survey Powered By Qualtrics
