# Supplementary material for: A randomised experiment of health, cost and social norm message frames to encourage acceptance of swaps in a simulation online supermarket
Source: PLoS One. 2021 Feb 17;16(2):e0246455. doi: 10.1371/journal.pone.0246455 (PMC7888673; doi:10.1371/journal.pone.0246455)
Supplement: S2 File — (DOCX) [file pone.0246455.s003.docx]

S2 File. list of product categories from within which lower-energy alternatives were identified for swaps.

Shelf

1. Sweet Biscuits
2. British & Tex Mex
3. Crisps & Snacks
4. Pasta
5. Fresh Beef
6. Continental Cheese
7. Fresh Pork
8. Sweets
9. Pickles
10. Mayonnaise & Salad Cream
11. Frozen pies & Quiches
12. Fresh Soup
13. Chocolate
14. Fresh Chicken
15. Cream
16. Sandwich Fillers
17. Mexican
18. Prepared Salad
19. Food Cupboard Free From
20. Frozen Fish
21. Seeded Bread
22. Cooked & Continental Meats
23. Tinned Fish & Seafood
24. Vegetarian & Healthy
25. Kids Snacking
26. Yoghurts
27. Fresh Lamb
28. Fresh Bacon & Gammon
29. Trifles Cheesecakes & Sundaes
30. Jams & Sweet spreads
31. Cereal Bars
32. Dried fruit & Nuts
33. Small Cakes
34. Counter - Fishmonger
35. Savoury Biscuits
36. Potato & Veg Side Dishes
37. Cheddar Cheese
38. Coleslaw & Dressed Salads
39. Counter - Butchers
40. Counter - Cheese
41. Counter - Delicatessen Meats
42. Nuts
43. Fresh Speciality Bread
44. Frozen Meat Free & Vegetarian
45. Pies & Quiche
46. Frozen Chips
47. Cheese Sliced & Grated
48. Soup
49. Fresh Cream Desserts
50. Sausages
51. Healthy Eating Cheese
52. Frozen desserts
53. Tinned Meat
54. Fresh Fish
55. Cheese Spreads & Snacks
56. Frozen Vegetables
57. Asian Groceries
58. Relishes & Chutneys
59. Indulgent Desserts
60. Desserts
61. Speciality Groceries
62. Frozen Ready Meals
63. Counter - Delicatessen Pies Savouries & Olives
64. Instant Snacks
65. Pulses
66. Fresh White Bread
67. Fresh Sweet Treats
68. Frozen Potatoes
69. Individual Chocolate Desserts
70. Healthier Cakes
71. Dips & Dressings
72. Prepared Meals
73. Croissants Brioche & Pastries
74. Rice
75. Antipasti
76. Flapjacks & Traybakes
77. Large Cakes
78. Tea Cakes Fruit Loaves & Scones
79. Tortes & Tarts
80. Lollies Bars & Cones
81. Dessert cases bases & Fillings
82. Fresh Pasta & Pasta Sauce
83. Bread Roll
84. Vegetables
85. Ice cream tubs
86. Sponges Pies & Puddings
87. Kosher Groceries
88. Indian
89. Italian
